# Impact of mouse model tumor implantation site on acquired resistance to anti-PD-1 immune checkpoint therapy

**DOI:** 10.3389/fimmu.2022.1011943

**Published:** 2023-01-10

**Authors:** Morgane Denis, Doriane Mathé, Manon Micoud, Pierre-Antoine Choffour, Chloé Grasselly, Eva-Laure Matera, Charles Dumontet

**Affiliations:** ^1^ Univ Lyon, Université Claude Bernard Lyon, INSERM 1052, CNRS 5286, Centre Léon Bérard, Centre de Recherche en Cancérologie de Lyon, Lyon, France; ^2^ R&D Department, Antineo, Lyon, France; ^3^ Hematology Department, Hospices Civils de Lyon, Lyon, France

**Keywords:** orthotopic, subcutaneous, anti-PD-1, MC38, preclinical model

## Abstract

**Introduction:**

The use of tumor subcutaneous (SC) implantations rather than orthotopic sites is likely to induce a significant bias, in particular, in the field of immunotherapy.

**Methods:**

In this study, we developed and characterized MC38 models, implanted subcutaneously and orthotopically, which were either sensitive or rendered resistant to anti-PD1 therapy. We characterized the tumor immune infiltrate by flow cytometry at baseline and after treatment.

**Results and Discussion:**

Our results demonstrate several differences between SC and orthotopic models at basal state, which tend to become similar after therapy. These results emphasize the need to take into account tumor implantation sites when performing preclinical studies with immunotherapeutic agents.

## Introduction

The discovery of immune checkpoint inhibitors (ICI) has deeply modified treatment in several cancer indications. These therapies enhance the activity of immune cells against tumors by impeding the immunoparesis induced by tumor cells. ICI targeting the PD-1/PD-L1 axis have shown significant antitumor activities in several tumor types (1–9). However, a majority of patients do not respond to therapy and a majority of those who are initially sensitive to ICI will eventually relapse. Understanding the mechanisms of resistance to ICI thus represents a major issue. In the case of primary resistance to ICI, access to patient samples is fairly straightforward. Conversely obtaining longitudinal samples of primarily sensitive and secondarily resistant patients is far more challenging, explaining the scarcity of data regarding secondary resistance mechanisms in the clinic. The best described acquired resistance mechanisms are the overexpression of alternative ICI such as TIM3 on immune cells or PD-L1 on tumor cells, the dysfunction of the presentation of the antigen by MHC I or the mutations of genes such as JAK1/2 (10–13).

The majority of murine syngeneic models are resistant to anti PD1/PDL1 therapies. However, there are very few models of secondary resistance to these compounds (14). To address this issue we have developed syngeneic models of resistance to ICI and found that the development of the resistant phenotype is associated with strong molecular and immunological heterogeneity (15). Another methodological difficulty is the fact that most tumor implantations in mice are performed subcutaneously rather than orthotopically. Since the tumor immune microenvironment (TIME) is critical in the case of ICI therapy, it is likely that the site of tumor implantation will have an impact on the nature and functionality of the tumor immune infiltrate. The development of well characterized orthotopic murine models of sensitivity and resistance to ICI may thus be expected to be better correlated with the situation encountered in patients than subcutaneous (SC) models.

Colorectal cancer (CRC), which is the third most prevalent type of neoplasia, was initially found to be poorly sensitive to ICI therapy, with a response rate of 5% (16, 17). However, response rates were found to be much higher when patients with microsatellite-instability-high (MSI-H) or mismatched repair-deficient phenotypes were considered. Additionally the Immunoscore has been suggested to help select patients with a higher probability of response to ICI therapies (18). There remains a unmet need to better modelize the impact of orthotopic implantation which is expected to be associated with a specific immune infiltrate, exposure to microbiota and ability to disseminate to liver, which is the most common site of metastases in the clinic (19).

To explore the impact of the implantation site in mouse colorectal tumor models we chose to compare subcutaneously and orthotopically implanted MC38 colorectal tumors, which were analyzed for their immune microenvironment and sensitivity to anti-PD1 therapy. We also developed resistant variants for both implantations and compared the alterations of the tumor immune microenvironment associated with acquisition of resistance. As presented in this manuscript, the major differences observed according to implantation site may be a major confounding factor in the preclinical modelization of ICI therapy.

## Materiel and methods

### Mouse cell line culture

MC38 cell line was obtained from Kerafast (CVCL_B288). Cell line was negative for mycoplasma assays. Murine colon cancer MC38 cells were cultured in DMEM medium (GibcoTM, 41966-029) with 10% fetal bovine serum (GibcoTM, A3160802), 100 U/mL penicillin and streptomycin (GibcoTM, 15140122). Cells were incubated in a humidified incubator with 5% CO_2_ at 37°C.

### Establishment of subcutaneous and orthotopic resistant models

MC38 cells were injected in 4-5 weeks female C57Bl/6 mice (Charles River Laboratory, 000664), For the SC model, 5.10^6^ cells of exponentially growing cultures were diluted in 0.2 mL of PBS (Gibco, 140040-091) and injected SC into the left flank. The tumor volume was measured every three days (length x width) with a caliper. The tumor volume was determined using the formula: 4/3 x π x r^3^. When the tumor volume reached 150 mm^3^, mice were randomized and received first treatment of anti-PD-1 (BioXCell, RMP1-14, BE014, RRID: AB_10949053, 12.5 mg/kg per week, intraperitoneal (IP)).

For the orthotopic model, a tumor established from a SC implantation was removed. A piece of it was grafted onto the cecum of the mice. Mice were treated with buprenorphine (Axience, 03760087151893), 30 min before tumor implantation. Mice were anesthetized with isoflurane (3%). Mice are shaved on their stomachs on the left side and then disinfected with betadine using sterile cotton pads. A first incision is made using a sterile 5mm SC scalpel. A 2nd 3mm incision is made on the peritoneum to find the organ as well as possible. The organ will be lifted using sterile forceps, the piece of tumor MC38 previously cut into 2mm sized pieces was transpierced with a thread (mono filament type PDS 5/0 crimped with a needle (22mm needle) and implanted onto the cecum. Abdominal wound and skin were closed with a 5/0 and 3/0 suture mono filament respectively (20). The mice are randomized based on their weight and receive 48 hours after the operation their first anti-PD-1 treatments (BioXCell, RMP1-14, BE014, RRID: AB_10949053, 12.5 mg/kg per week, IP).

To establish the resistant models, tumors obtained from mice with initial responses to anti-PD-1 implanted SC or orthotopically, were serially reimplanted into new groups of naive mice and treated once a week to maintain selection pressure as described above. At each passage, three naïve mice were implanted with tumor fragments and treatment was initiated once the tumor reached 150 mm^3^ or 48h after implantation for SC or orthotopic implantation, respectively. The most aggressive tumor was selected for reimplantation. At least 5 and 7 passages were necessary to induce acquired resistance for SC and orthotopic implantation sites, respectively. We will refer to sensitive models as wild type (WT) and to resistant models as anti-PD-1-R for those resistant to anti-PD-1 mAbs. All mice were raised in an SPF environment with free access to standard food and water. Experiments using C57Bl/6 mice were submitted to and approved by the Animal Ethics Committee CECCAPP of Lyon.

### Immune cells panel - Aurora spectral Cytek

Immunophenotyping of the TIME was performed at baseline and after therapy. A first analysis was performed when the tumor volume reached approximately 200mm^3^ for the SC implantation and four days after surgery for the orthotopic counterpart. A second analysis was performed four days after the second weekly treatment, both for SC and orthotopic models. Samples were acquired on a Cytek^®^ Aurora flow cytometer with SpectroFlo^®^Software (Cytek^®^ Biosciences). For all experiments, to digest tumor we used the gentle MACS Octo Dissociator (130-096-427, Miltenyl Biotec) with mouse tumor dissociation kits (130-096-730, Miltenyl Biotec). After filtration through a 100 µm filter (130-110-917, Miltenyl Biotec) and wash, cells were stained with a viability dye marker (Zombie UV, Biolegend, 423108) and blocked with anti-CD16/32 antibody (Biolegend, 101320) according to the manufacturer’s instructions. Cells were stained with the fluorescently labelled antibodies in the dark for 30 min at 4°C After surface staining, cells were fixed and permeabilized using BD Cytofix/Cytoperm kit (BD, 554714), then labeled with F4/80, FoxP3, Granzyme B, CD206 and T-bet in the dark for 30 min at 4°C (**Table 1**). FlowJoV10 software (BD) was used for analyses as described previously (15) and GraphPad Prism software was used for statistical analysis (ANOVA with Bonferroni post-test). Experiments were performed twice for SC models, and once for orthotopic models. Gating strategies are described in **Supplementary Figure 1**.

**Table 1:** Key resources.

REAGENT or RESOURCE	SOURCE	IDENTIFIER	RRID
Antibodies for Flow cytometry experiment			
CD25	BD	565134	AB_2744344
CD45	BD	564279	AB_2651134
CD8	BD	750024	AB_2874242
I-A/I-E	BD	748846	AB_2873249
CD172a	BD	741593	AB_2871002
NKp46	BD	612805	AB_2870131
CD11c	BD	749038	AB_2873432
CD38	BD	740489	AB_2740212
CD19	BD	747332	AB_2872036
CD64	BD	741024	AB_2740644
Ly6C	BD	553104	AB_394628
CD62L	BD	565261	AB_2739138
CD206	Biolegend	141732	AB_2565932
PD-1	Biolegend	109121	AB_2687080
CD4	BD	563106	AB_2687550
CD44	Biolegend	103037	AB_10900641
F4/80	Biolegend	123147	AB_2564588
T-bet	Biolegend	644810	AB_2200542
Ly6G	Biolegend	127616	AB_1877271
Viability UV Zombie	Biolegend	423108	
CD3	Thermo	58-0032-82	AB_11217479
Granzyme B	Thermo	MHGB05	AB_10373420
CD11b	Thermo	48-0112-82	AB_1582236
FoxP3	Thermo	50-5773-82	AB_11218868
CD49b	Thermo	15-5971-82	AB_2573070
CD24	Thermo	46-0242-82	AB_1834425
SiglecH	Thermo	63-0333-82	AB_2784853
PD-L1	Thermo	25-5982-82	AB_2573509
CD107a+	Thermo	47-1071-82	AB_2848363
Antibodies *In vivo*			
InVivoMab anti-mouse CD279 (PD-1) (clone RMP1-14)	Bioxcell	BE0146	AB_10949053
Experimental Models: Organisms/Strains			
C57BL/6 mice	Charles Rivers	000664	
Experimental Models: Cell Lines			
MC38	Kerafast	CVCL_B288	CVCL_B288
Medium culture			
DMEM medium	Gibco™	41966-029	
Fetal bovine serum	Gibco™	A3160802	
Antibiotics (Pen/Strep)	Gibco™	15140122	
Critical Commercial Assays			
Mouse Tumor Dissociation Kit	Miltenyl Biotec	130-096-730	
MACS SmartStrainers	Miltenyl Biotec	130-110-917	
Chemical, Peptides and Recombinant Proteins			
BD Cytofix/Cytoperm	BD	554714	
Red blood cell lysis solution	Miltenyi Biotec	130-094-183	
Immunohistochemistry experiments			
**Hematoxylin**	Diapath	CO283	
Eosin	Merck	1.15926	
Superfrost+ slide	Epredia	J1800AMNZ	

### Immunochemistry

After fixation, MC38 tumors were dehydrated and impregnated in the LEICA ASP300 machine. After inclusion, samples were cut to 3 μm and mounted on a Superfrost+ slide (Epredia, J1800AMNZ). Slides were dewaxing with methyl and alcohol and rehydrated. Hematoxylin (Diapath, CO283) was added for 30 sec and slides were rinsed with water. Eosin (Merck, 1.15926) was added for 1min 30sec and slides were rinsed with water. Finally, slides were dehydrated and cover slipped. Slides were then scanned on the slide scanner Zeiss Axio Scan.Z1 to obtain representative images.

### Statistics

Errors bars relate to SEM unless indicated in all figure legends. Using GraphPad Prism (V9), Mann Whitney t-test or two-way ANOVA statistical tests, with Bonferroni *post hoc* test. Statistical significances are indicated as follows: *p<0.05, **p<0.01, ***p<0.001, ****p<0,0001.

## Results

### Subcutaneous and orthotopic implantations of MC38 are sensitive to anti-PD-1

**Figure 1:**
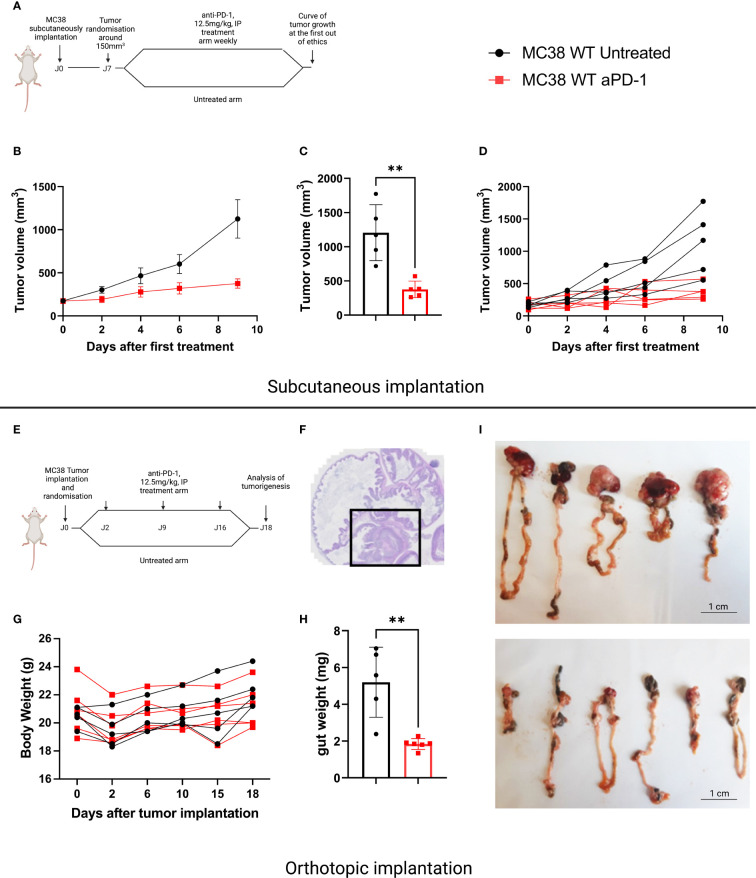
Sensitivity to anti-PD-1 treatment on the MC38 subcutaneous versus orthotopic implantation models. **(A)** Treatment administration for C57Bl/6 mice SC implanted with a MC38 tumor fragment. The mice were randomized when the tumor volume reached 150 mm3 then treated or not with anti-PD-1 (BioXCell, BE0146, RRID: AB_10949053, 12.5 mg/kg per week, intraperitoneal (i.p.)). **(B)** Tumor growth in mice SC grafted with MC38, untreated or treated with anti-PD-1. **(C)** Individual values of the last measurement point of the mice having received treatment or not. **(D)** Individual curves of tumor growth in mice SC grafted with MC38, untreated or treated with anti-PD-1. **(E)** Treatment administration for C57Bl/6 mice orthotopically implanted with a tumor fragment of MC38. **(F)** Representative image of MC38 tumor implanted in the cecum on D4. **(G)** Individual curves representing the weight change in grams of mice orthotopically grafted with MC38, untreated or treated with anti-PD-1. **(H)** Individual values of the weight of the intestines at D18 of the mice having received treatment or not. **(I)** Photographs of untreated and treated intestines grafted with orthotopic MC38 at D18. Data represent mean tumor volume and error bars represent ± SEM. Results are representative of three independent experiments, n = 5 per groups, **p < 0.01, Mann Whitney t-test.

The murine MC38 colorectal cancer cell line is widely used by the scientific community since it demonstrates sensitivity to anti-PD-1. However, this observation has mainly been documented when this cell line is implanted SC. We compared sensitivity to anti-PD-1 treatment of tumors developed by SC and orthotopic implantation. For SC implantation, we chose a classic administration schedule, namely weekly intraperitoneal administrations of 12.5mg/kg anti-PD-1 antibodies, initiated once tumor volumes were 150mm^3^ (**Figure 1A**). As described in the literature, we obtained a partial response following the anti-PD-1 treatment compared with the control mice (**Figures 1B–D**). In the case of orthotopic implantation, we observed that tumors grew more aggressively than mice implanted subcutaneously. We therefore chose to treat mice 48 hours after implantation, with a once weekly administration of 12.5 mg/kg (**Figure 1E**). Mice were weighed and the abdomens palpated daily (**Figure 1F**). In the orthotopic study, all mice were euthanized once one of the mice had reached a predefined endpoint. This allowed us to observe that orthotopically implanted MC38 models demonstrated sensitivity to anti-PD-1 therapy (**Figures 1G–I**).

### Subcutaneous and orthotopic implantations of MC38 become resistant to anti-PD-1

**Figure 2:**
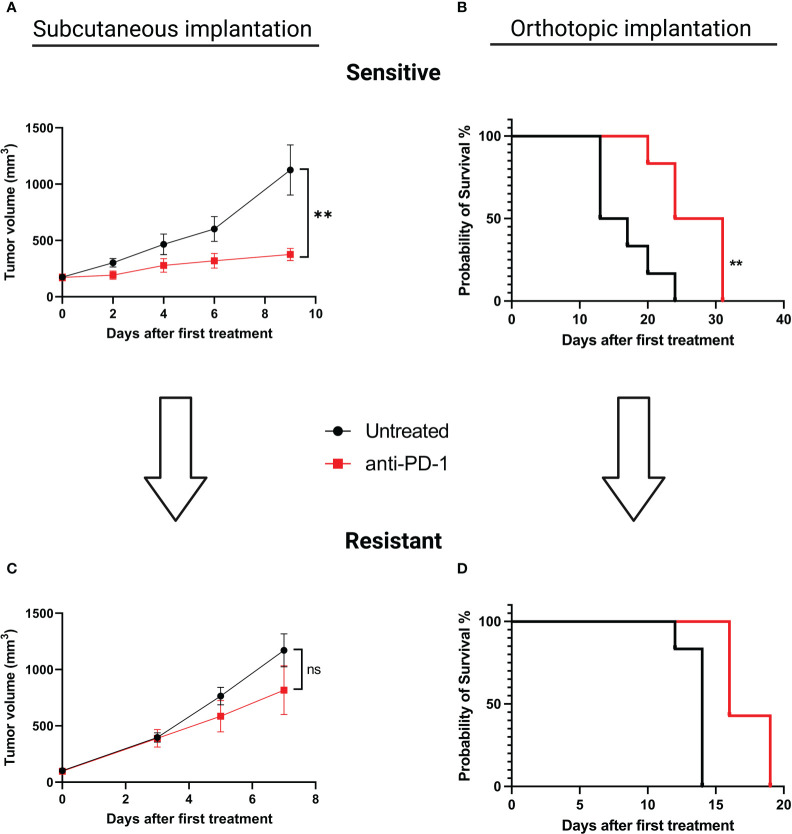
Induction of acquired resistance to anti-PD-1 therapy in the SC versus orthotopically implanted MC38 model. For the SC model implantation, 5.10^6^ tumor cells were injected in each animal. When tumors reached a volume of 150 mm3, mice were randomized and treated with aPD-1 (BioXCell, 12.5 mg/kg per week, i.p.). For the orthotopic model implantation, fragments of tumors were implanted in the cecum. Two days after surgical implantation mice were randomized according to body weight and treated or not with aPD-1 (BioXCell, 12.5 mg/kg per week, i.p.). For both types of implantations, fragments of tumors displaying a primary response to aPD-1 were then implanted into new groups of tumor-naive mice and treated once a week to maintain selection pressure. At least five and seven passages were necessary to induce acquired resistance for SC and orthotopic implantations, respectively. **(A)** Tumor growth in mice grafted with MC38 subcutaneously, untreated or treated with anti-PD-1. **(B)** Overall survival of mice grafted with orthotopic MC38, untreated or treated with anti-PD-1. **(C)** Tumor growth in mice grafted SC with MC38 model that had been rendered resistant to anti-PD-1. **(D)** Overall survival of mice grafted orthotopically with MC38 model that been rendered resistant to anti-PD-1. Data shown represent mean tumor volumes and error bars represent ± SEM. Results are representative of three independent experiments for the SC model and two independent experiments for the orthotopic model, n = 5 per groups, **p < 0.01, Mann Whitney t-test. ns: non significant.

To explore alterations associated with the acquisition of an ICI-resistant phenotype we chose to induce acquired resistance in our SC and orthotopic models. We performed serial reimplantation with repeated selection pressure with anti-PD-1 therapy. After five passages for the SC model and seven passages for the orthotopic model, tumor growth was similar in treated and untreated mice, confirming the acquisition of a resistant phenotype. (**Figures 2A, B** for sensitive and **2C, 2D** for resistant models, respectively). In relationship with anti-PD-1 sensitivity, we will refer to “MC38 sensitive” versus “MC38 resistant”.

### Implantation sites affect basal immune microenvironment in MC38 sensitive model

**Figure 3:**
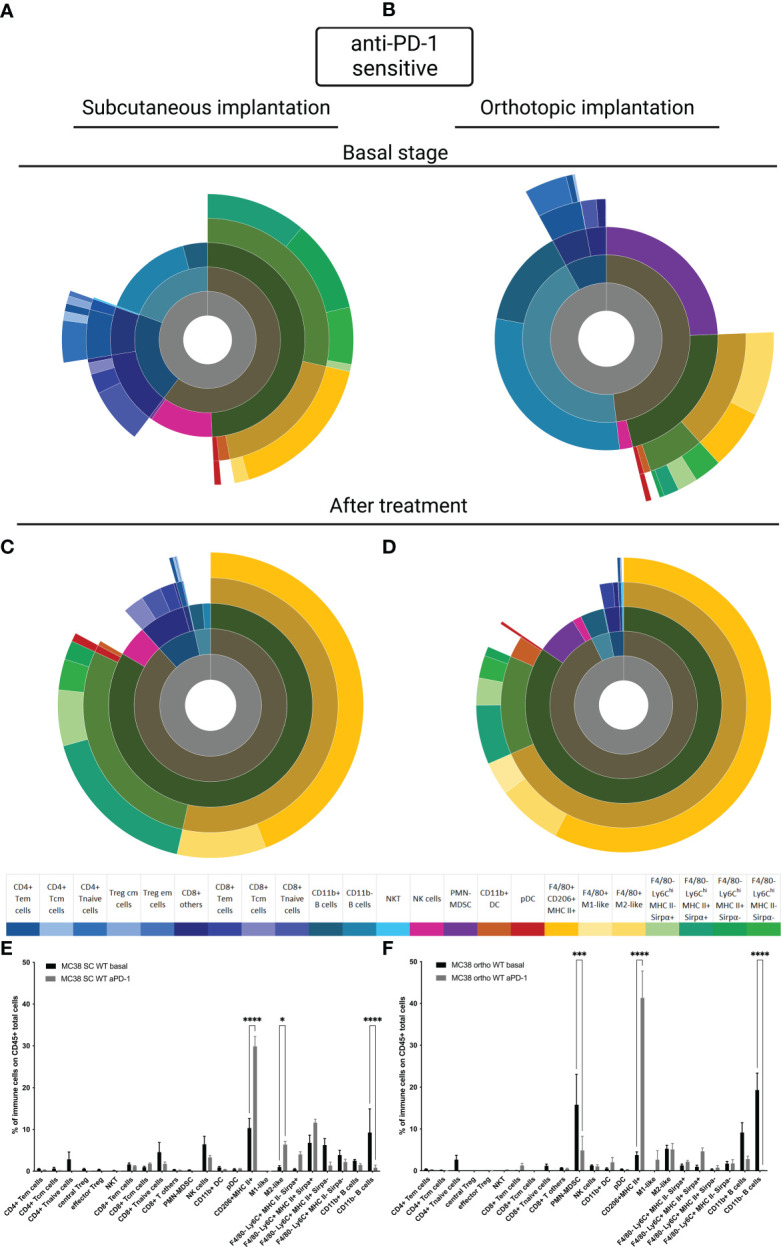
MC38 sensitive tumor TIME at basal and after anti-PD-1 therapy. Flow cytometry experiments were performed when tumors reached 200 mm^3^ or two days after implantation for basal stage for SC and orthotopic implantation respectively and four days after second treatment for both. **(A–D)** Sunburst plots showing the proportion of CD45+ immune infiltration. **(A)** Basal stage for SC implantation, **(B)** Basal stage for orthotopic implantation, **(C)** Anti-PD-1 treated group for SC implantation, **(D)** Anti-PD-1 treated group for orthotopic implantation. **(E, F)** Histograms shown mean of percentage values and error bars are SEM of basal versus under treatment for **(E)** SC model and **(F)** Orthotopic model. Flow cytometry plots represent a pool of five tumor samples. Significant decreases and increases were assessed by a two-way ANOVA statistical test, with Bonferroni *post hoc* test. Statistical significances are indicated as follows: *p < 0.05, ***p < 0.001, ****p < 0,0001.

To understand whether tumor implantation had an impact on the TIME, we performed the immunophenotyping of MC38 sensitive tumor models at basal state and after treatment for both sensitive models implanted subcutaneously or orthotopically. For the SC model, we observed the same results reported in the literature, in particular at basal state in the MC38 sensitive model implanted subcutaneously, a large proportion of CD11b+ cells were detected and an equivalent proportion of lymphoid B and T cells and NK cells were identified (**Figure 3A**) (15, 21). In the MC38 sensitive model implanted orthotopically we observed an extensive proportion of B cells and polymorphonuclear-myeloid-derived suppressor cells (PMN-MDSC) (**Figure 3B**). Moreover other myeloid cells, T cells and NK cells were less represented in the immune tumor microenvironment of MC38 implanted orthotopically than in the SC localization (**Figure 3B**). After treatment, the TIME tended to be similar between tumor implantation sites (**Figures 3C, D**), with a significant down-regulation of B cells and an upregulation of F4/80+CD206+MHC-II+ cells in both SC and orthotopic sensitive MC38 tumors exposed to anti-PD1 antibody (p<0,0001, **Figures 3E, F**). Moreover, we detected a significant down-regulation of the PMN-MDSC infiltrate induced by therapy in the MC38 sensitive orthotopic tumor (p<0,001, **Figure 3F**). These data show that exposure to anti-PD1 therapy may induce a homogeneous immune infiltrate, independently on the site of implantation. This observation could be explained by the fact that the post-therapeutic infiltrate is determined by the impact of therapy on immune cell recruitment or activation rather than by the composition of the preexisting immune infiltrate.

### Immune microenvironment in MC38 resistant anti-PD-1 model depending on the implantation site

**Figure 4:**
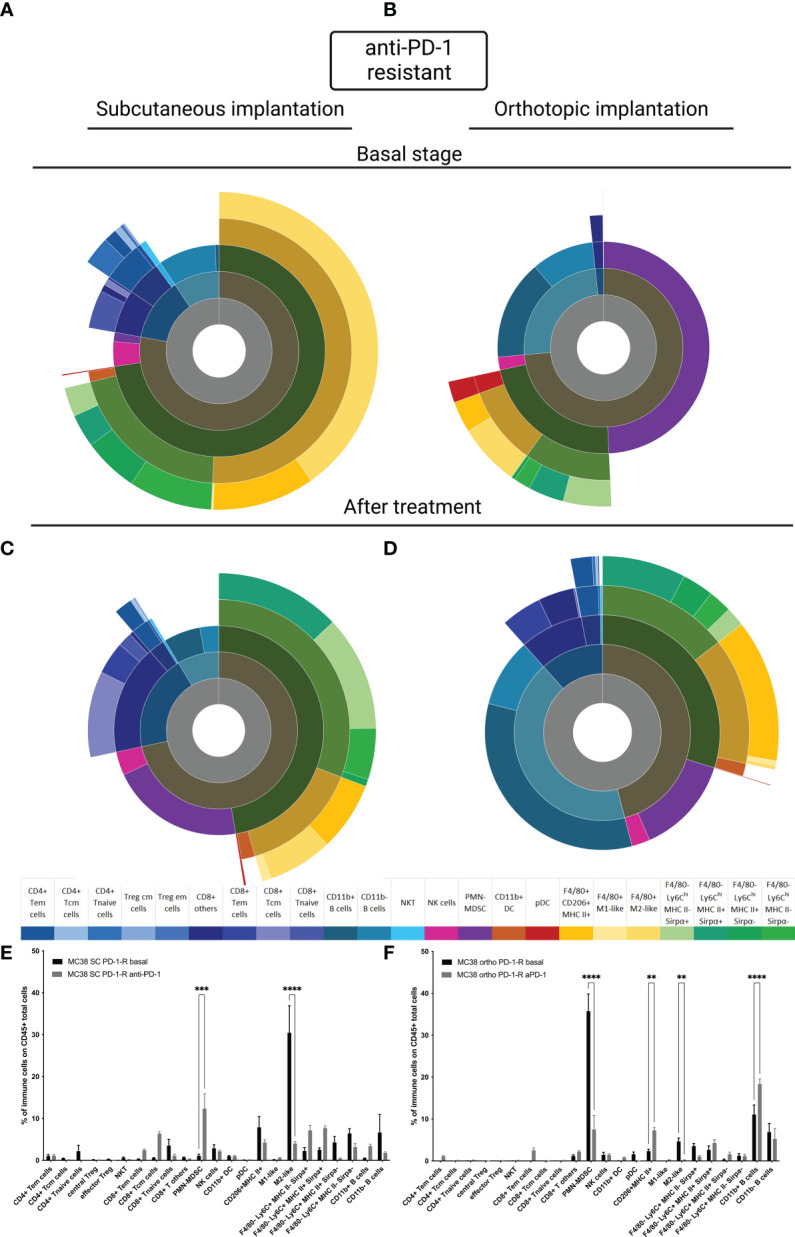
MC38 resistant tumor to anti-PD-1 TIME at basal and under selection pressure of therapy. Flow cytometry experiments were performed when tumors reached 200 mm^3^ or two days after implantation for basal stage for SC and orthotopic implantation respectively and four days after second treatment for both. **(A–D)** Sunburst plots showing the proportion of CD45+ immune infiltration. **(A)** Basal stage for SC implantation, **(B)** Basal stage for orthotopic implantation, **(C)** Anti-PD-1 treated group for SC implantation, **(D)** Anti-PD-1 treated group for orthotopic implantation. **(E, F)** Histograms shown means of percentage values and error bars are SEM of basal versus under treatment for **(E)** SC model and **(F)** Orthotopic model. Flow cytometry plots represent a pool of five to ten tumor samples. Significant decreases and increases were assessed by a two-way ANOVA statistical tests, with Bonferroni *post hoc* test. Statistical significances are indicated as follows: **p < 0.01, ***p < 0.001, ****p < 0,0001.

To better understand whether the site of implantation influences the acquisition of resistance to anti-PD-1, we carried out a study of TIME on our MC38 resistant models at the basal state and under selection pressure. Firstly, at the basal state, as for the sensitive model, the TIME is extremely different depending on the implantation site (**Figures 4A, B**). In the SC MC38 resistant model, as previously described, we found a higher proportion of TAM M2-like cells compared to the sensitive model (15) but also compared to the orthotopic resistant model. In the orthotopic model, at the basal state, we also observed an absence of infiltration by T cells as well as a strong infiltration of pDC. Moreover, we detected a very strong infiltration of PMN-MDSC and B cells.

Under selection pressure, we detected a significant down-regulation of M2-Like cells (p<0,0001, **Figures 4C, E** and p<0,01 **Figures 4D, F**) and an up regulation of CD11b+ B cells, in both models (**Figures 4C–F**) (22). Focusing on PMN-MDSC, our results showed an increase of the population in the SC model and a decreased in the orthotopic model (p<0,001 and p<0,0001 respectively). This inversed trend lead to an equivalent final proportion in both models (**Figures 4C–F**). All these data allowed us to identify immune cells with a potential impact, on the acquired resistance to anti-PD-1.

## Discussion

The influence of the implantation site on the composition of the tumor immune infiltrate in preclinical models is poorly understood and studied, although it is likely to impact on the sensitivity to immunotherapeutic agents and mechanisms of resistance to ICI therapy. There is clear evidence that the organ in which a tumor originates influences the composition of the TIME and influences response to therapy (23–25). A recent study reported that translating preclinical observations from mice to men remains unreliable, with rates ranging between 0 and 100% (26). Several causes may explain this observation. To understand whether the recruitment of immune cells in the tumor is different depending on the site of implantation, we performed an immunophenotyping of TIME. Our study demonstrated that in the MC38 sensitive SC and orthotopic models, TIME composition is strikingly different at the basal state but tends to become similar over time when anti-PD-1 is administered. Our results suggest that the high level of infiltration by immunosuppressive cells such as TAM may block the response mediated by CD8+ T lymphocytes (27). This result is supported by the study by Abou-Elkacem et al. which elegantly demonstrated that the efficacy of anti-PD-1 treatment is dependent on the presence of CD8+ T cells, but also that the depletion of TAMs improved the antitumor activity of treatment (24). In the orthotopic model, the strong increase in dendritic cells observed after treatment suggests the establishment of an immune response mediated by CD8+ T lymphocytes, which might not yet be effective. Taken together these data suggest that recruitment of immune cells under the effect of anti-PD-1 treatments is similar, whether MC38 tumors are implanted SC or orthotopically and despite differences in TIME at the basal state.

However, after acquisition of resistance, the immune tumor landscape is very different. In particular, the strong presence of PMN-MDSCs in the two models suggests a predominant role of these cells in the acquisition of resistance to anti-PD-1. It has been reported that MC38 sensitive tumors implanted SC do not have a detectable PMN-MDSC infiltrate (19). Clearly, our results show that in sensitive orthotopic tumors and in resistant models, MC38 tumors are infiltrated by PMN-MDSC. However, PMN-MDSC become predominant in the SC resistant model compared to the sensitive model. This is in keeping with our previous observation that the combination anti-PD1 therapy with an anti-Ly6G antibody reverses resistance to anti-PD-1 (15). However, for the orthotopic model, the proportion of PMN-MDSC diminished after treatment in both site of implantation but the proportion remains higher in the resistant model compared to the sensitive model. Longitudinal immune characterization obtained in the two resistant models suggest that PMN-MDSCs play a role in the induction of secondary resistance but not in the same time lapse. Moreover, in the orthotopic resistant model, we no longer detected T lymphocytes at the basal state, as well as very few under treatment pressure. This result suggests that the resistance to anti-PD-1 therapy also involves the absence of T cells in the TIME. Recently it was reported that IL-17 mediates neutrophil recruitment and triggers Neutrophil Extracellular Traps (NETs) in the tumor microenvironment. The presence of NETs in the tumor microenvironment correlates with CD8 T cell exclusion, and IL-17 blockade increased sensitivity to ICIs (25). In addition, NETs coat tumor cells and shield them from Natural Killer cells and CD8 T cell-mediated cytotoxicity. NET inhibition has been shown to sensitize tumors to ICIs (26).

Moreover, B cell content which was increased in the resistant models after therapy could also be involved. Very few publications have focused on B cell implication in the context of anti-PD-1 therapy, as opposed to T cells. A recent publication showed that the presence or absence of B cells had no impact on the efficacy of anti-PD-1 (27). It is described in the literature that CD11b+ B cells have a higher ability to drive T cell proliferation than CD11b- B cells (28). However, CD11b+ B cells may also spontaneously secrete IL-10 and suppress T cells activation (29). Moreover, the implication of B cells such as Breg on acquired resistance to anti-PD-1 deserve to be studied more extensively. B cells play a pivotal role in several diseases and our study suggest that B cells may also be involved in acquired resistance to anti-PD-1.

To conclude, TIME in SC and orthotopically implanted tumors differ significantly for several types of immune cells. It is possible that cells that induce resistance are more easily and abundantly recruited in the orthotopic model compared to the SC model. Our results emphasize the need to consider the site of implantation in preclinical modeling of immunotherapy.

## Data availability statement

The raw data supporting the conclusions of this article will be made available by the authors, without undue reservation.

## Ethics statement

The animal study was reviewed and approved by Animal Ethics Committee CECCAPP of Lyon.

## Author contributions

MD designed the experiments. DM, MM, P-AC, CG, E-LM assisted MD with experiments. MD analyzed data. CD advised with methods and theory of experiments. MD wrote the manuscript in consultation with CD and CD supervised the project. All authors contributed to the article and approved the submitted version.

## Supplementary material

Supplementary Figure 1 Gating strategy for FACS analysis.
