# Supplementary figures and images for: Impact of mouse model tumor implantation site on acquired resistance to anti-PD-1 immune checkpoint therapy
Source: Front Immunol. 2023 Jan 10;13:1011943. doi: 10.3389/fimmu.2022.1011943 (PMC9872099; doi:10.3389/fimmu.2022.1011943)

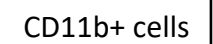

Supplement: Supplementary Figure 1 — Gating strategy for FACS analysis. [file DataSheet_1.pdf]
